# Etiology and mode of presentation of chronic liver diseases in India: A multi centric study

**DOI:** 10.1371/journal.pone.0187033

**Published:** 2017-10-26

**Authors:** Partha S. Mukherjee, Sreenivas Vishnubhatla, Deepak N. Amarapurkar, Kausik Das, Ajit Sood, Yogesh K. Chawla, Chundamannil E. Eapen, Prabhakar Boddu, Varghese Thomas, Subodh Varshney, Diamond Sharma Hidangmayum, Pradip Bhaumik, Bhaskar Thakur, Subrat K. Acharya, Abhijit Chowdhury

**Affiliations:** 1 Liver Foundation, West Bengal. Kolkata, West Bengal, India; 2 Department of Biostatistics, All India Institute of Medical Sciences, Ansari Nagar, New Delhi, India; 3 Bombay Hospital & Medical Research Centre, Mumbai, India; 4 Department of Hepatology, School of Digestive and liver Diseases, Institute of Post Graduate Medical Education & Research, Kolkata, India; 5 Department of Gastroenterology, Dayanand Medical College & Hospital, Ludhiana, Punjab, India; 6 Department of Hepatology, Post Graduate Institute of Medical Sciences, Chandigarh, India; 7 Department of Hepatology, Christian Medical College, Vellore, India; 8 Department of Gastroenterology, Osmania General Hospital, Afzalgunj, Hyderabad, Telangana, India; 9 Department of Gastroenterology, Calicut Medical College, Kozhikode, Kerala, India; 10 Department of Surgical Gastroenterology, Bhopal Memorial Hospital and Research Centre, Bhopal, Madhya Pradesh, India; 11 Catholic medical centre hospital, Koirengei, Imphal East, Manipur, India; 12 Department of medicine, Agartala Govt Medical College, Agartala, Tripura, India; 13 Department of Gastroenterology and Human Nutrition, All India Institute of Medical Sciences, Ansari Nagar, New Delhi, India; 14 Indian Institute of Liver and Digestive Sciences, Sitala (East), Jagadishpur, Sonarpur, 24 Pgs(S), Kolkata, India; Saint Louis University, UNITED STATES

## Abstract

There is a paucity of health policy relevant data for chronic liver disease from India, impeding formulation of an interventional strategy to address the issue. A prospective, multicentric study to delineate the etiology and clinical profile of chronic liver disease in India is reported here. A centrally coordinated and monitored web-based data repository was developed (Feb, 2010 to Jan, 2013) and analyzed. Eleven hospitals from different parts of India participated. Data were uploaded into a web based proforma and monitored by a single centre according to a standardized protocol. 1.28% (n = 266621) of all patients (n = 20701383) attending the eleven participating hospitals of India had liver disease. 65807 (24·68%) were diagnosed for the first time (new cases). Of these, 13014 (19·77%, median age 43 years, 73% males) cases of chronic liver disease were finally analyzed. 33.9% presented with decompensated cirrhosis. Alcoholism (34·3% of 4413) was the commonest cause of cirrhosis while Hepatitis B (33·3%) was predominant cause of chronic liver disease in general and non-cirrhotic chronic liver disease (40·8% out of 8163). There was significant interregional differences (hepatitis C in North, hepatitis B in East and South, alcohol in North-east, Non-alcoholic Fatty Liver Disease in West) in the predominant cause of chronic liver disease. Hepatitis B (46·8% of 438 cases) was the commonest cause of hepatocellular Cancer.11·7% had diabetes. Observations of our study will help guide a contextually relevant liver care policy for India and could serve as a framework for similar endeavor in other developing countries as well.

## Introduction

Chronic liver diseases (CLD) cause significant morbidity and mortality worldwide. Multiple etiological factors lead to a similar clinico-pathological syndrome in CLDs, although the rates of progression and clinical course may be different [1,2]. Mortality data is most often used to assess the disease burden and there had been a 46% increase in CLD mortality in the world between 1980 to 2013, underscoring the emerging public health importance of CLD. Most of this increase in CLD mortality has been reported from the low and low-middle income (LMIC) countries of Asia and Africa [3]. It is intriguing to note that most countries in these region have very poor vital events reporting systems, indicating that the current data could underestimate the existing situation and complimentary approaches are needed to assess the overall impact of CLDs on health systems [4,5,6,7].

Low and middle income countries (LMIC) are experiencing demographic and epidemiologic transition in disease burden. India is one of the epicenters of this change [8,9,10]. Clinical and vital events reporting in India is still fragmentary and usage of electronic medical records in hospitals is just beginning to take shape. In such a context of organized resources for meaningful data, policy framework suffers along with planning and allocation of resources.

We report here an analysis based on 13014 newly diagnosed cases of CLD, enrolled over a three year period (2010–2013) from 11 tertiary care centers across the country. Specific objectives of the study were to capture: (a) at what clinical stage of disease do CLD patients seek clinical care (b) what are the different etiologies and how do they vary in different parts of the country and (c) broad social and demographic issues influencing access to healthcare facilities in India.

## Patients and methods

**Fig 1:**
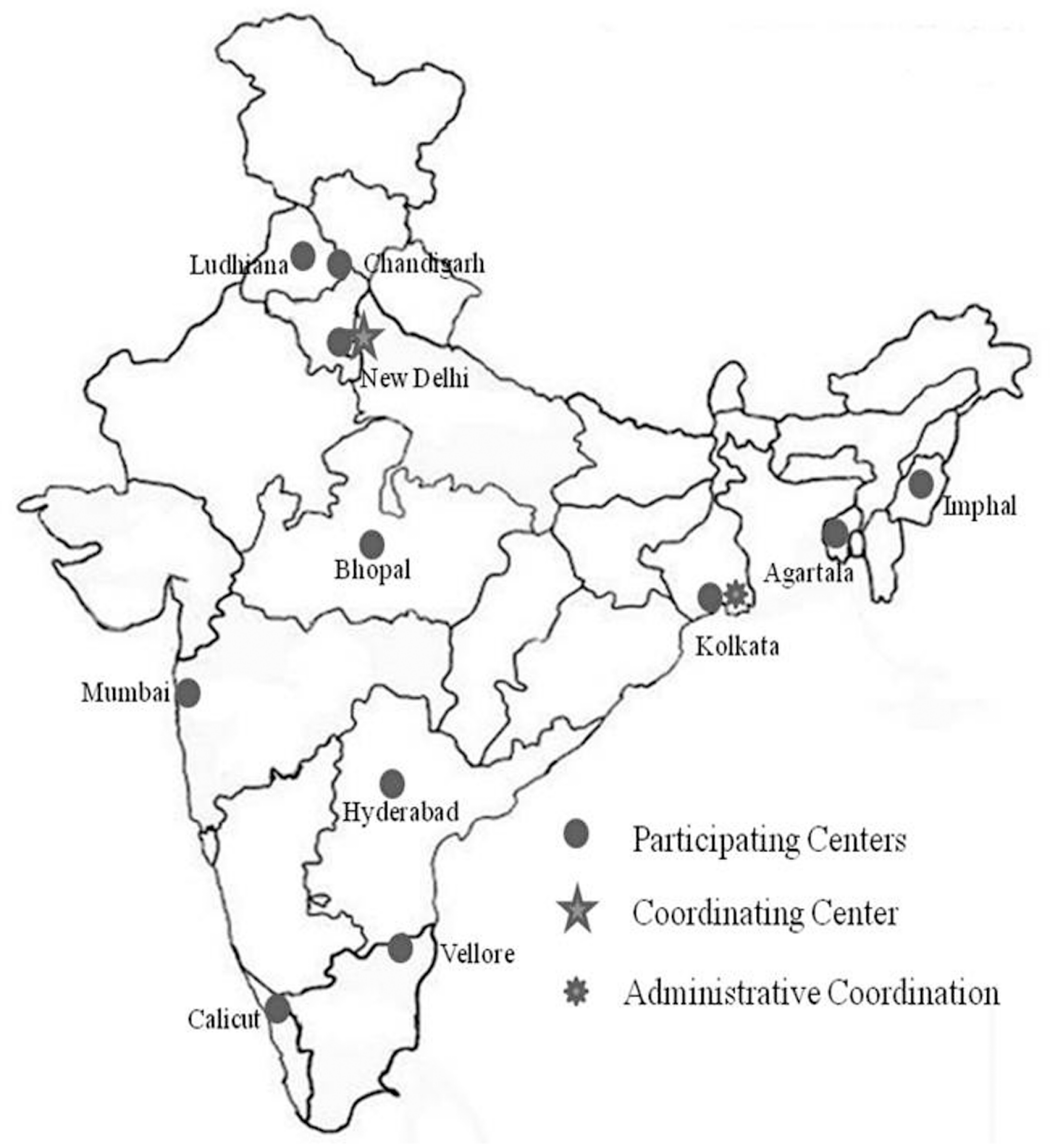
Layout of study network across the country.

“HCV:- the Indian face” was a prospective, hospital based, liver disease data repository that included liver disease of all etiologies. Data were collected over a defined period (April 2010 to March 2013) according to a uniform protocol in 11 (eleven) centers spread across the country (Fig 1). Each case was clinically assessed by a physician before enrollment.

### Study design and data management

In order to achieve a representative data and capture the regional differences existing in the country, the participating centers were selected from five geographically different regions of the country (Fig 1). The centers were at: North India (Delhi, Chandigarh, Ludhiana), South India (Hyderabad, Vellore, Calicut), West (Mumbai), central (Bhopal), East (Kolkata) and northeast India (Agartala, Manipur). All centers were tertiary care hospitals. Centers in Ludhiana and Vellore were Private Teaching Hospitals. Center in Bhopal was Government non-teaching hospital. Centers in Mumbai and Manipur were private hospitals. Rest of the tertiary care centers were government teaching institutes. The single monitoring and coordinating center was at Delhi. Patients were assigned to the region according to their residence.

The structured proforma, used for data collection, was developed through a workshop where all the investigators agreed upon the uniform diagnostic definitions of the clinical phenotypes (S2 Appendix). It was pilot tested and then applied. The questionnaire administration, data procurement was done by trained interviewers, under supervision of clinicians. This data was later uploaded to a web-based repository at the end of each day. The data once uploaded could be accessed only from a single central port (SV) which was responsible for coordination and monitoring. Data quality control checks and clarification on ambiguities were dealt with by periodic visits by central team, through e-mail and telephone calls.

### Approval by ethics committee

All patients provided written informed consent to participate in this observational study. Study was approved by ‘Institutional Ethics Committee’ of Institute of Post Graduate Medical Education & Research, Kolkata, India in a meeting held on 27^th^ March, 2010 complying with the acceptable international standard (Declaration of Helsinki) (S3 Appendix).

The full name of the ethics committee was ‘Institutional Ethics Committee, Institute of Post Graduate Medical Education & Research, 244, A. J. C. Bose Road, Kolkata-20’ (website: http://www.ipgmer.gov.in/researchoversightcommittee.html). Study was conducted in other participating centers based on this ethical approval.

### Definitions used in this study

1. *Cirrhosis of liver (LC*): Cirrhosis of liver with portal hypertension was diagnosed based on standard clinical feature (presence of ascites), radiological evidences (shrunken liver, dilated portal vein with Periportal or other collaterals) and endoscopic evidence (presence of esophageal/gastric/ectopic varices and/or portal hypertensive gastropathy). [1] Liver biopsy was performed in patients without these features and who were willing for the procedure and having no contraindication for liver biopsy.
  Cirrhosis of liver once diagnosed was classified according to standard Child-Pugh scores (scores 5–6 for Child class A, 7–9 for Child class B and 10–15 for Child class C). [1]
2. *Non cirrhotic chronic liver disease (NCCLD)*: This was defined as an inclusive category for all patients that presented with history and/or evidence of chronic liver dysfunction in the form of ultrasound proven hepatomegaly and/or persistent abnormality (more than 6 month duration) in liver function test (elevated liver enzymes and/or jaundice) but did not qualify to be labeled as cirrhosis or hepatocellular carcinoma.
3. *Hepatocellular carcinoma (HCC);* Diagnosed by standard criteria [11].
4. *Below poverty line (BPL)*: was defined according to criteria laid down by planning commission of government of India using monthly per-capita consumption expenditure (MPCE) in terms of per capita income per day of $ 2·14 (Rs. 32·33) and $ 3·10 (Rs. 47) in rural and urban areas, respectively [12].
5. *Literacy*: Literacy as per National Literacy Mission was defined as ‘acquiring the skills of reading, writing and arithmetic and the ability to apply them to one's day-to-day life’ [13]. Whether the patient was able to use vernacular language accordingly in his daily life was assessed during interview.

### Etiological evaluation

Etiology of liver disease was ascertained by the clinician investigator at each center according to standard clinical protocols (S1 Appendix).

### Statistics and analysis

**Fig 2:**
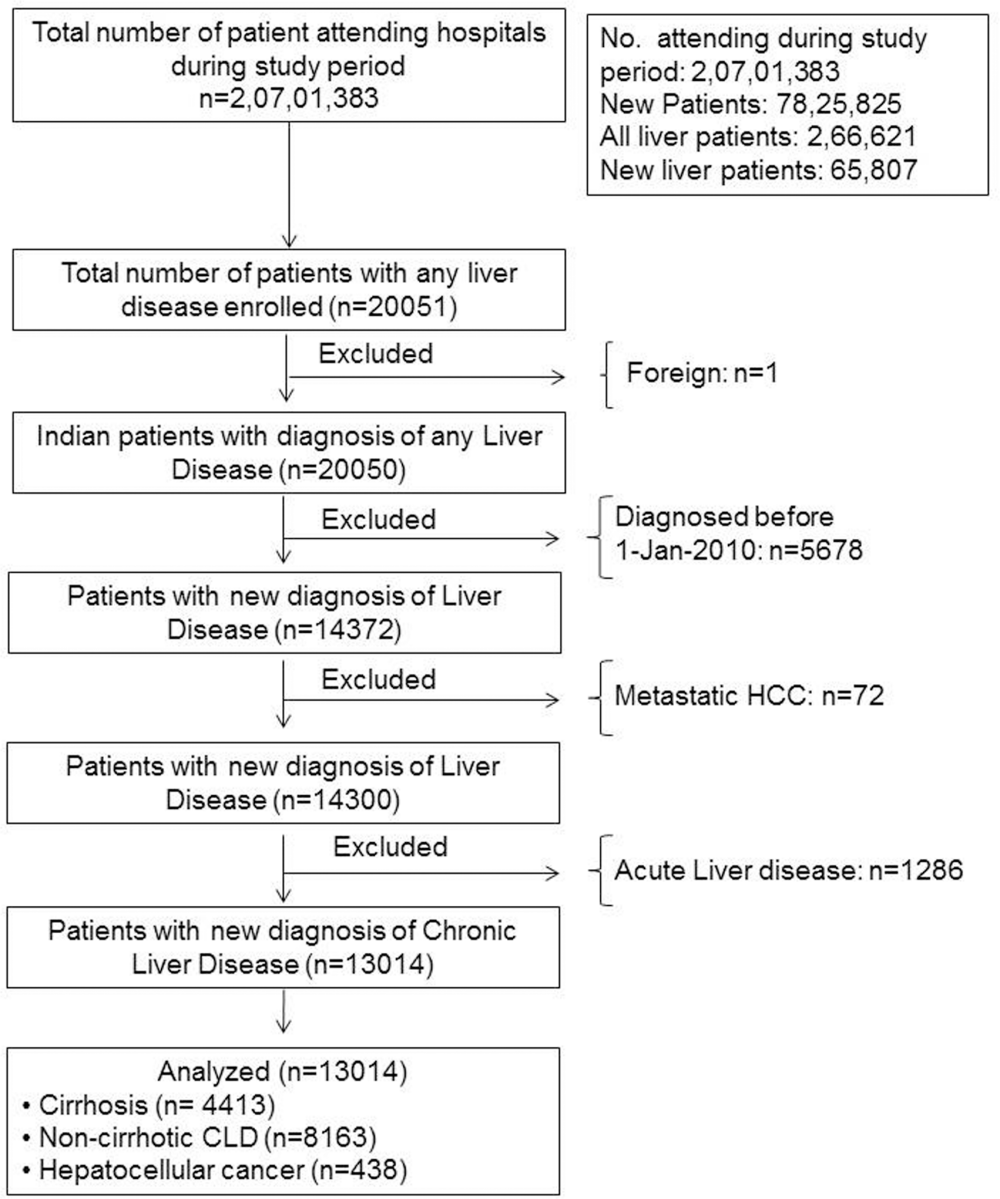
Study design.

Final analysis was done on the patients with newly diagnosed CLD only (n = 13014) (Fig 2). Categorical and discrete variables are presented in percentage. Continuous variables are presented as mean±SD (standard deviation) or Median (range) wherever applicable. Comparisons between the groups were done by using ANOVA. A p value less than 0·05 were taken as significant. Multiple pair-wise comparisons among the regions of the country were done for socio-demographic parameters and etiological distribution. A logistic regression analysis was done to see the relationship between socio-economic parameters and presence of cirrhosis at the time of diagnosis of chronic liver disease. All analyses were implemented on Stata 14.1.

## Results

Patient inclusion plan is summarized in Fig 2. A total of 20701383 patients attended the participating hospitals during the study period i.e. February, 2010 to January, 2013. Liver disease in any form was diagnosed in 266621 patients (1·28%). Of these, 65807 (24·68%) were newly diagnosed to have liver disease. Out of these, 14372 (21·8% of new liver disease) were enrolled into the study. We excluded metastatic cancer (n = 72) and acute liver disease cases (n = 1286). Final analysis was done on the newly diagnosed CLD (n = 13014) which comprise 19·77% of all newly diagnosed liver disease patients (n = 65807) (Fig 2).

### Access related issues: Social and healthcare facility usage profile

Overall about half of the patients were resident of urban areas (n = 6564, 50·4%). The urban representation was comparatively higher in the central, western and north-east region of the country (88%, 85·9% and 69·2%, respectively; Table 1). Majority of the patients belonged to the above poverty line (APL) social class with regional variations. Patients with below poverty line (BPL) status varied from 40·7% in the eastern region to 2·1% in western region (Table 1 and S1 Table). Patients with more severe forms of liver disease i.e. cirrhosis and HCC were more dependent on public healthcare system (41·2% and 49·3% in cirrhosis and HCC, respectively vs 25·5% in non-cirrhotic liver disease; Table 2), irrespective of their economic status. Comparison between the patients above and below poverty line (APL vs BPL) shows that HCV is the second most frequent etiology in APL group, whereas, alcohol is in BPL group. Both cirrhosis and decompensation at diagnosis of liver disease and Diabetes as comorbidity were higher in BPL group. Patients in BPL group avail government health care facility more frequently and reside comparatively at farther distance from nearest health care facility (S2 Table).

**Table 1:** Characteristics of the chronic liver disease patients with regional variation.

Characteristic		All Patients (n = 13014)	Region						P
			North (n = 4342)	East (n = 3385)	South (n = 1854)	West (n = 1818)	North-East (n = 504)	Central (n = 1111)	
Urban dweller; n (%)		6555 (50·4)	1862 (42·9)	1115 (32·9)	687 (37·1)	1562 (85·9)	351 (69·6)	978 (88·0)	<0·001
Patients without literacy; n (%)		1147 (8·8)	473 (10·9)	397 (11·7)	161 (8·7)	17 (0·9)	29 (5·8)	70 (6·3)	<0·001
Patients below poverty line; n (%)		2715 (23·4)	337 (11·2)	1368 (40·7)	643 (35·2)	39 (2·2)	184 (36·7)	144 (13·0)	<0·001
Age at diagnosis (Years)	Mean±SD	42·8±14·4	41·1±14·3	41·7±14·4	43·0±14·9	46·3±14·3	44·4±11·6	46·3±13·8	<0·001
	Median (Range)	43 (1–95)	40 (1–87)	42 (1–86)	43 (2–95)	46 (1–87)	45 (15–77)	47 (2–90)	<0·001
Duration of disease before diagnosis (Months) Median (Range)		0·13 (0–34·2)	0·57 (0–34·2)	0·33 (0–32·6)	0 (0–25·4)	0·30 (0–32·5)	0·88 (0–26·7)	0 (0–29·4)	<0·001
Etiology n (%)	HBV related^$^	4336 (33·3)	1205 (27·8)	1621 (47·9)	750(40·5)	320(17·6)	150 (29·8)	290 (26·1)	
	HCV related^$^	2806 (21·6)	1951 (44·9)	317 (9·4)	202 (10·9)	147 (8·1)	131 (26·0)	58 (5·2)	
	Alcohol related	2253 (17·3)	475 (10·9)	579 (17·1)	564 (30·4)	312 (17·2)	161 (31·9)	162 (14·6)	
	NAFLD related	1664 (12·8)	300 (6·9)	87 (2·6)	57 (3·1)	719 (39·6)	17 (3·4)	484 (43·6)	
	Others	2021 (15·5)	456 (10·5)	785 (23·2)	286 (15·4)	326 (17·9)	49 (9·7)	119 (10·7)	
Diabetes, n (%)		1524 (11·7)	376 (8·7)	290 (8·6)	222 (12·0)	413 (22·7)	45 (8·9)	178 (16·0)	<0·001
Current alcohol user, n (%)		2429 (18·7)	695 (16·0)	509 (15·0)	483 (26·0)	416 (22·9)	134 (26·6)	192 (17·3)	<0·001
Prevalence of HCC, n (%)		438 (3·4)	216 (5·0)	130 (3·8)	26 (1·4)	20 (1·1)	7 (1·4)	39 (3·5)	<0·001
Patients with cirrhosis; n (%)		4413 (33·9)	1160 (26·7)	1565 (46·2)	640 (34·5)	548 (30·1)	258 (51·2)	242 (21·8)	<0·001
Distance to nearest health-care facility (km); Median (IQR)		2 (0–5)	0 (0–4)	3 (2–8)	9 (3–55)	0 (0–0)	4 (2–13)	2 (2–3)	<0·001
Availing government health care facility; n (%)		4115 (31·6)	1182 (27·2)	1440 (42·5)	597 (32·2)	29 (1·6)	214 (42·5)	653 (58·8)	<0·001
Referral pattern	Teaching institute	2477 (19·0)	1042 (24·0)	698 (20·6)	410 (22·1)	69 (3·8)	242 (48·0)	16 (1·4)	<0·001
	Non-teaching Inst	1283 (9·9)	385 (8·9)	517 (15·3)	290 (15·6)	4 (0·2)	17 (3·4)	70 (6·3)	<0·001
	Private practitioners	5326 (40·9)	1661 (38·2)	1240 (36·6)	1068 (57·6)	1124 (61·8)	123 (24·4)	110 (9·9)	<0·001
	Self-referral	3238 (24·9)	1235 (28·4)	907 (26·8)	78 (4·2)	154 (8·5)	113 (22·4)	751 (67·6)	<0·001
	Friend/Relative	661 (5·1)	19 (0·4)	23 (0·7)	8 (0·4)	453 (24·9)	3 (0·6)	155 (13·9)	<0·001
	Other	29 (0·2)	0 (0·0)	0 (0·0)	0 (0·0)	14 (0·8)	6 (1·2)	9 (0·8)	<0·001

$ Dual viral infections counted twice, both in HBV & HCV

**Table 2:** Characteristics of the chronic liver disease patients according to pattern of the disease.

Characteristic		All Patients (n = 13014)	Cirrhosis (n = 4413)	Non-cirrhotic chronic liver disease (n = 8163)	Hepatocellular cancer (n = 438)	P
Male, n (%)		9504 (73·0)	3469 (78·6)	5666 (69·4)	369 (84·2)	<0·001
Age at diagnosis (Years)	Mean ± SD	42·8±14·4	47·7±13·4	39·6±13·9	54·4±13·8	<0·001
	Median (Range)	43 (1–95)	48 (1–88)	39 (1–90)	56 (4–95)	<0·001
Duration of disease before enrolment (Months); Median (Range)		0·13 (0–34·2)	0·4 (0–34·2)	0·03 (0–32·6)	0·32 (0–24·9)	<0·001
Etiology, n (%)	HBV related	4336 (33·3)	800 (18·1)	3331 (40·8)	205 (46·8)	<0·001
	HCV related	2806 (21·6)	762 (17·3	1979 (24·2)	65 (14·8)	<0·001
	Alcohol related	2253 (17·3)	1512 (34·3)	699 (8·6)	42 (9·6)	<0·001
	NAFLD related	1664 (12·8)	77 (1·7)	1587 (19·4)	0 (0·0)	<0·001
	Other	2021 (15·5)	1281 (29·0)	613 (7·5)	127 (29·0)	<0·001
Diabetes, n (%)		1524 (11·7)	746 (16·9)	704 (8·6)	74 (16·9)	<0·001
Current alcohol user, n (%)		2429 (18·7)	1270 (28·8)	1080 (13·2)	79 (18·0)	<0·001
Distance to nearest health-care facility (km); Median (IQR)		2 (0–5)	2 (0–5)	2 (0–6)	3 (1–5)	<0·001
Availing government health care facility; n (%)		4115 (31·6)	1820 (41·2)	2079 (25·5)	216 (49·3)	<0·001
Referral pattern; n (%)	Teaching institute	2477 (19·0)	1166 (26·4)	1226 (15·0)	85 (19·4)	<0·001
	Non-teaching Institute	1283 (9·9)	664 (15·0)	556 (6·8)	63 (14·4)	<0·001
	Private practitioners	5326 (40·9)	1307 (29·6)	3856 (47·2)	163 (37·2)	<0·001
	Self-referral	3238 (24·9)	1091 (24·7)	2027 (24·8)	120 (27·4)	<0·001
	Friend/Relative	661 (5·1)	178 (4·0)	476 (5·8)	7 (1·6)	<0·001
	Other	29 (0·2)	7 (0·2)	22 (0·3)	0 (0·0)	<0·001
Cirrhosis Status; n (%)	Known		4266			
	Compensated		26 (0·6)			
	Decompensated		4240 (99·4)			

### Demographic patterns

Median age of presentation with CLD was 43 years. Regional variation in age of presentation was noted. Regions with predominant viral etiologies for liver disease (northern, eastern and southern regions) had relatively lower median age at diagnosis (40, 42 and 43 years, respectively). Whereas, regions with NAFLD as predominant etiology (western and central region) show higher median age at diagnosis (46 and 47 years, respectively) (Table 1 and S1 Table). On the other side, patients with HBV related CLD were younger at diagnosis (median 36 years) in comparison to those with alcohol and NAFLD related disease (median 46 and 45 years, respectively) (Table 3). In addition, an increasing gradient of median age at the time of diagnosis was noted from non-cirrhotic CLD to cirrhosis and HCC (Table 2). Men predominated in all clinical patterns of CLD as well as across the etiologies (Tables 2 and 3).

**Table 3:** Etiology-specific profile of chronic liver disease.

Characteristics		All patients (n = 13014)	HBV* (n = 4336)	HCV* (n = 2806)	Alcoholic (n = 2253)	NAFLD (n = 1664)	Misc (n = 2021)	P Value
Male, n (%)		9504 (73·0)	3238 (74·7)	1903 (67·8)	2218 (98·4)	987 (59·3)	1208 (59·8)	<0·001
Age at diagnosis (years)	Mean±SD	42·8±14·4	38·1±14·3	43·8±13·8	46·8±10·6	45·2±13·2	45·1±17·3	<0·001
	Median (Range)	43 (1–95)	36 (1–90)	44 (1–87)	46 (20–85)	45 (1–88)	46 (1–95)	<0·001
Diabetes, n (%)		1524 (11·7)	263 (6·1)	265 (9·4)	283 (12·6)	335 (20·1)	383 (19·0)	<0·001
Current alcohol user, n (%)		2429 (18·7)	477 (11·0)	308 (11·0)	1394 (61·9)	161 (9·7)	100 (4·9)	<0·001

* Dual viral infections counted twice, both in HBV & HCV; Misc Miscellaneous; SD Standard Deviation

### Disease patterns

Overall, 33·9% (4413 out of 13027) of the patients had cirrhosis. Proportion of cirrhosis was significantly higher in eastern (1565 out of 3386, 46·2%) and north-eastern region (258 out of 510, 51·2%) (Table 1 and S1 Table). Almost all of the cirrhotics had decompensation at the time of diagnosis (4240 out of 4266, 99·4%) (Table 2).

### Etiological profile

HBV was the commonest cause of CLD. There was significant regional variation in etiology across the country (Table 1 and S1 Table). HCV was the commonest cause in the northern region (1951 out of 4343 patients, 44·9%). HBV was predominant in the east and south (1621 out of 3386, 47·9% and 750 out of 1855, 40·6% in eastern and southern regions, respectively) and second most frequent etiology in rest of the country. Alcohol was in the north-eastern regions of the country (161 out of 510, 31·6%), closely followed by HBV and HCV (Table 1). NAFLD was in western and central regions of the country (719 out of 1822, 39·5% and 484 out of 1111, 43·6%, respectively). HCV genotype 3 was predominant in north, east, west and north-east regions and genotype 4 was in south and central regions (Table 4).

**Table 4:** Regional variation of genotypes of chronic Hepatitis C^Δ^.

Genotype; n (%)	Region						Total
	North	East	South	West	Central	North-East	
1	130 (18·3)	16 (18·2)	7 (13·5)	18 (24·3)	1 (3·0)	3 (11·1)	175 (17·8)
2	5 (0·7)	4 (4·6)	0 (0·0)	1 (1·4)	0 (0·0)	0 (0·0)	10 (1·0)
3	462 (65·0)	37 (42·0)	15 (28·8)	49 (66·2)	10 (30·3)	19 (70·4)	592 (60·1)
4	114 (16·0)	31 (35·2)	30 (57·7)	6 (8·1)	22 (66·7)	5 (18·5)	208 (21·1)
Total; n (%)	711 (100·0)	88 (100·0)	52 (100·0)	74 (100·0)	33 (100·0)	27 (100·0)	985 (100·0)

Δ Genotyping was done in 985 HCV cases

Alcohol emerged as the most common etiology of cirrhosis (1512 out of 4413, 34·3%), while HBV was commonest cause in the non-cirrhotic CLD (3331 out of 8163, 40·8%) and HCC (205 out of 438, 46·8%) (Table 2).

Diabetes was present in 11·7% (1524 out of 13014) of newly diagnosed CLD patients at the time of diagnosis. Overall, cirrhotic patients had higher proportion of diabetics than non-cirrhotics (746 out of 4413, 16·9% and 704 out of 8163, 8·6%) (Table 2). Diabetes as a comorbidity was more prevalent in western and central regions (414 out of 1822, 22·7% and 178 out of 1111, 16%, respectively) where NAFLD was also commonest cause of CLD (Table 1). Apart from NAFLD, patients with alcohol related CLD had higher proportion of diabetics (283 out of 2253, 12·6%) (Table 3). Description of different etiologies included in ‘other’ etiology group in the Table 1 is provided in S3 Table.

### Hepatocellular cancer (HCC)

HCC was found in 3·4% of the patients (438 out of 13014 patients) with CLD at the time of diagnosis (Table 1). Prevalence of HCC was highest in northern region (216 out of 4342, 5%) and lowest in western and southern regions (20 out of 1818, 1·1% and 26 out of 1854, 1·4%, respectively) (Table 1). Patients with HCC were significantly older at presentation than those with cirrhosis without HCC (Median age 56 vs 48, in HCC vs Cirrhosis without HCC, respectively, p<0·001) (Table 2). HBV was most frequent etiology (205 out of 438, 46·8%) followed by HCV (65 out of 438).

## Discussion

The present study was designed to ascertain clinical, etiological and access related features pertaining to chronic liver diseases in India. These are of relevance for formulating policy and addressing issues of health system preparedness including allocation of resources for liver diseases in India. Important observations of this study include the finding that at least one third of the CLDs present at a remarkably advanced stage of decompensated cirrhosis in India, there was significant regional differences regarding predominant etiology within the country, diabetes was frequent comorbidity and socio economic disparities characterize access to available care facilities. Hepatitis B vaccine was introduced in the Universal Immunization Program (UIP) of 10 states of India in the year 2007–08 [14]. We believe these findings from a country wide network of hospitals will provide a fair framework for future data resources for comparisons and are also likely to be informative for planning liver disease care in India.

We demonstrate based on data that chronic liver diseases present fairly late in Indian hospitals, including after onset of decompensation, in about one third of the patients. In the context of the resource constraint health systems like India and many other LMICs, this observation of a relatively late presentation of a fairly large segment of CLD patients is of particular concern [15,16]. Care for liver disease is maximally effective if instituted early. Chronic liver diseases are characterized by an indolent but inexorably progressive course in the absence of etiology specific interventions. Such course can remain clinically unapparent for long periods and can become evident only after liver failure sets in, often with dysfunction of other vital organs [1,2]. In view of this, screening and building awareness about liver diseases are considered to be important strategic interventions in liver disease policy and needs to be priorities in Indian context. Improving liver disease awareness, risk factor detection and improved professional education might all be considered important interventions to impact on this scenario.

The etiological profile of CLD in India, as brought out in our data, highlights the epidemiological transition that the country is passing through. HBV remained the most common cause of CLD overall, while alcoholism was the primary runner for cirrhosis. Our study also brings out important regional differences in viral hepatitis with HCV “pockets” in north and northeast India while HBV was predominant etiology in east and south India and second most common etiology in rest of the country. While this is in general agreement with the available data from India, it mandates the need for assessing contextual peculiarities while planning health care resource and target allocations. This is more so in the light of the global strategic plan for viral Hepatitis of WHO, where emphasis has been put in addressing the contextual situation in each country [17,18,19,20].

Significance of alcoholism and diabetes remain the two emerging features of our study that needs mention. Alcohol was the most common cause of cirrhosis and one fifth of the patients of all etiologies were current alcohol consumers while 12% patients had diabetes. There is a surge of alcohol related morbidity at a global scale and the Indian scenario is similar, with earlier age at alcoholism, an increasing per capita intake as well as trends of increasing “at risk” drinking as accompaniments of urbanization, globalization and a westernized lifestyle that the country is adopting [21]. Side by side, NAFLD in south Asia has been shown to lead to significant CLD at relatively low adiposity [22]. Consistent with this, our study shows that diabetes and thereby NAFLD is coming up as a significant etiology and comorbidity having negative impact on the course and outcome of the liver diseases of other etiologies, as has been observed in other developing countries [23].

We found that 1·2% of all hospital attendances in Indian hospitals were due to CLD alone and the fact that only one fourth of all liver disease patients attending hospitals were newly diagnosed. This compares favorably with estimates of death due to cirrhosis in India i.e. age standardized death rate 23·6 per 100,000 population and 2% of death due to all causes. CLD mortality figures in India are increasing progressively since 1980 while that of China, the other Asian country with a large population remain stationary and is even showing downward trends in mortality [17,24].

We observed significant socioeconomic disparities in health facility usage and liver disease care seeking in India. The proportion of rural, poor and illiterate patients included in our study was less than that existing in Indian society as a whole. These raise concerns of access to available care. Social and ethnic disparities have been demonstrated to be important impediments of equitable liver disease care organization in other populations, including Africa and USA [25,26]. Such issues assume increasing importance with progressively increasing corporate participation in tertiary, including advanced liver disease care, in India. Increasing participation of the government in liver disease care, like that has been done with the Hepatitis C policy in the Punjab state, might be helpful in improving this scenario and resolve access issues [27].

The strength of our data is in the meticulous clinical assessment of each case before enrollment that avoided misclassification and representation of entire country using country-wide network. The strategy that we used in the current study was different from other burden estimate studies which mostly relied on acquisition of data from available resources, including national surveillance systems, insurance data, electronic medical records etc. Study specific web-based data repository with a central monitoring system circumvented the disadvantage of not having structured electronic data record system in most of the Indian hospitals.

Despite having large number of cases and wide geographical base of our study, our study had several limitations. Although centers were included from different parts of the country, this was based more on feasibility and known expertise in liver disease care, rather than based on a population based representative sampling. Despite the strengths of the study discussed earlier, it must be acknowledged that larger and more representative samples are needed to improve the generalizability of the findings to the country as a whole. In addition, while by strategy the study was simplistic to be able to capture the broad picture of liver disease in India, more analytical inputs were needed for delineation of clinical details that we targeted to achieve.

## Conclusion

Present study provide a much needed and useful sketch of the clinical patterns, etiologies, regional differences and overall trends in access as well as relevant care utilization of CLD in India. In addition, the profile described here forms a benchmark for any comparisons in the future.

## Supporting information

S1 Appendix Protocol for etiological work up in patients with chronic liver disease. (DOCX)

S2 Appendix Proforma (database on liver diseases). (DOC)

S3 Appendix Approval letter from Institutional Ethics Committee. (PDF)

S1 Table Pair-wise comparison of regions (P values) for Table 1. (DOCX)

S2 Table Comparison of patients belonging to above and below poverty line. (DOCX)

S3 Table Break up of other etiologies mentioned in Table 1. (DOCX)
