# Supplementary material for: Etiology and mode of presentation of chronic liver diseases in India: A multi centric study
Source: PLoS One. 2017 Oct 26;12(10):e0187033. doi: 10.1371/journal.pone.0187033 (PMC5658106; doi:10.1371/journal.pone.0187033)
Supplement: S1 Appendix — (DOCX) [file pone.0187033.s001.docx]

**S1 Appendix**

**Protocol for etiological work up in patients with chronic liver disease:**

First step:

- Viral Markers (HBsAg, Anti-HCV),
- Assessment of alcohol intake (

Second step: If all negative in first step –

- Serum Ceruloplasmin, 24 hour urinary copper excretion test, Ophthalmological (Slit lamp) examination to exclude KF ring (For Wilson’s disease)
- Standard Autoimmune markers (ANA, ASMA, AMA)
- Metabolic work up for Non-alcoholic Steatohepatitis (NASH) – FBG, PPBG, Lipid profile

Third step: Liver biopsy in patients if following criteria is met –

- Patients without cirrhosis
- Patients with no contraindication for liver biopsy
- providing informed consent for the procedure
- Liver histology will help in diagnosis
- Liver histology will help in management plan including prognostication
