# Supplementary material for: Etiology and mode of presentation of chronic liver diseases in India: A multi centric study
Source: PLoS One. 2017 Oct 26;12(10):e0187033. doi: 10.1371/journal.pone.0187033 (PMC5658106; doi:10.1371/journal.pone.0187033)
Supplement: S2 Appendix — (DOC) [file pone.0187033.s002.doc]

# S2 Appendix

# Proforma (database on liver diseases)

# (To be filled while enrolling a patient for the first time into the study)

## IDENTIFICATION PARTICULARS

1. Centre ………………………………………………………………………….

Delhi-1 Kolkata-2 Chandigarh-3 Ludhiana-4 Calicut-5

Hyderabad-6 Bhopal-7 Manipur-8 Mumbai-9 Guwahati-10 Vellore-11

1. Serial Number ………………………………………………………….
2. Clinic Number …………………………………………………..
3. Date Registered in GE Dept. (dd/mm/yy) ………………………

## 5. Old or new case (1-Old 2-New) ………………………………………………

6. Date of this visit (dd/mm/yy) …………………………………..

## DEMOGRAPHIC DETAILS

1. Name of the patient
2. Age (Completed years, NK-99) ………………………………………………
3. Gender (Male-1, Female-2) ………………………………………………………..
4. Present Address:
5. Permanent Address:
6. State ……………………………………………………………………………

Andhra Pr.-01 Arunachal Pr.–02 Assam-03 Andam. & Nic.-04

Bihar-05 Chandigarh-06 Chattisgarh-07 Delhi-08

Goa-09 Gujarat-10 Haryana-11 Himachal Pr.-12

J.& Kasmir-13 Jharkhand-14 Karnataka-15 Kerala-16

Madhya Pr.-17 Maharashtra-18 Meghalaya-19 Mizoram-20 Nagaland-21 Orissa-22 Punjab-23 Rajasthan-24 Sikkim-25 Tamilnadu-26 Tripura-27 Uttaranchal-28 Uttar Pr.-29 West Bengal-30 Manipur-31 Lakshadweep-32

Daman, Diu-33 Dadra, Nagar Haveli-34 Pondicherry-35

1. Telephone number: …………………………….
2. Referred from: …………………………………………………………………….

Teaching Inst-1 Non-teaching Inst.-2 Pvt Practitioner-3 Self -4 Other (Specify)-5

1. Educational qualification ………………………………………………………….

Illiterate-1 Primary-2 Middle-3 Secondary-4 Higher secondary-5

College-6 Professional/PG-7 Not known-9

1. Approximate monthly family income ……………………………..

Enter monthly income if < 1 lakh

99998 if monthly income is > 1 lakh

99999 if monthly income is not known

**LIEFESTYLE INFORMATION**

1. Diet (Vegetarian-1 Non-vegetarian-2) …………………………………………….
2. Fast food taking (No-1 Yes-2) …………………………………………………….
3. Staple food (Rice-1 Wheat-2 Other-3) ……………………………………………
4. Do you smoke (No-1 Yes-2 Past smoker-3 Not Known-9) ……………………
5. If Yes, number smoked …………………………………………………….

If daily, 1 followed by the number of cigarettes per day

If weekly, 2 followed by the number of cigarettes per week

If monthly, 3 followed by the number of cigarettes per month.

If yearly, 4 followed by the number of cigarettes per year.

If non-smoker, enter 000

999 if Not Known

22. Duration of smoking in months (000 if no smoking) ………………………

1. Do you drink alcohol (No-1 Yes-2 Past consumer -3 NK-9) …………………
2. If Yes, amount (gms) consumed ………………………………………

If daily, 1 followed by the amount (gms) per day

If weekly, 2 followed by the amount (gms) per week

If monthly, 3 followed by the amount (gms) per month

If occasional, 4 followed by the amount (gms) per occasion

0000 if no alcohol consumption

9999 if Not Known

1. Main type of alcohol consumed ……………………………………………………

IMFL (Beer/Whiskey/Rum/Gin/Vodka/Wine) -1

Country (Neera/toddy/ arrack/ mahua/kala etc) -2

0 if no alcohol consumption

9 if Not Known

1. Duration of alcohol consumption in years (00 if no alcohol) …………………
2. Habit of using OTC drugs (No-1 Yes-2) ………………………………………..

If yes, enter duration (months), Enter 000 if no usage

a) Fever (crocin, paracetamol etc)……………………….

b) Pain Relievers/NSAIDS (Anacin, Disprin, Brufen etc)..

c) Cold and Cough (Expectorants/ D-cold etc) …………..

d) Anti-biotics ……………………………………………

e) Any other (specify) ……………………………………

**HEALTHCARE UTILIZATION DETAILS**

(To be confirmed with records wherever possible)

1. Received childhood immunization (No-1 Yes-2 NK-9) ………………………..
2. Received HBV vaccine (No-1 Yes-2 NK-9)……………………………………
3. Medical care mostly obtained from: Govt. Hosp-1 Pvt. Practitioner/Hosp-2 …….

Qualified alternative medical practitioner-3 Vaid/Hakim-4

1. Approx distance to nearest health-care facility (in Km, if < 1, enter 00) ………
2. Source of medicines used (Mostly purchased-1 Received free from Govt. hosp-2)
3. Number of episodes of major illness till date …………………………………..
4. Number of medical visits to any hospital/clinic till date
5. Total for all problems ……………………………
6. For liver related illness ………………………………
7. Number of hospitalizations (including emergency admission) till date
8. Total for all problems ……………………………….
9. For liver related illness ……………………………….
10. Number of hospitalizations on emergency basis till date ………………………….
11. Number of hospitalization on emergency basis for liver related illness till date ….

**HEALTHCARE COST DEATILS**

1. Approx. amount (Rs) spent for doctor’s fee / hospital fee till date
2. Total for all problems ………………………..
3. For liver related illness ………………………
4. Approximate amount (Rs) spent for procuring medicine till date

a) Total for all problems ………………………..

b) For liver related illness ……………………..

1. Approximate amount (Rs) spent for laboratory investigation till date
2. Total for all problems ……………………….
3. For liver related illness ………………………
4. Approximate amount (Rs) spent during hospitalization till date
5. Total for all problems ……………………….
6. For liver related illness ………………………
7. Approximate amount (Rs) spent for special procedures ……….

for liver related illness (EST/EVL/TACE etc)

1. Approximate amount (Rs) spent on antiviral drugs (if applicable)
2. Indirect cost (Total income lost due to illness) till date

(for transport, local conveyance, accommodation, food, salary loss etc. for the patient as well as accompanying persons)

**DURATION OF COMPLAINTS IN MONTHS**

**(Enter 000 if no complaint and 999 if NK/NA)**

1. Duration of Complaints of Jaundice………………………………………..
2. Duration of complaints of Weight loss…………………………………….
3. Duration of complaints of Pain …………………………………………….
4. Duration of complaints of Abdominal mass ……………………………….
5. Duration of complaints of Abdominal distension ………………………….
6. Duration of complaints of Fever ……………………………………………
7. Duration of complaints of UGI bleed ………………………………………
8. Duration of complaints of LGI bleed ………………………………………
9. Duration of complaints of Oedema …………………………………………
10. Duration of complaints of Anorexia ……………………………………….

# **PAST HISTORY (No-1 Yes-2 Not Known-9)**

1. H/o Bleeding ………………………………………………………………………
2. H/o BT ……………………………………………………………………………..
3. H/o Jaundice ……………………………………………………………………….
4. H/o Prolonged Illness ……………………………………………………………..
5. H/o Encephalopathy ………………………………………………………………
6. H/o Surgery. ……………………………………………………………………….
7. H/o Abdominal distension ………………………………………………………..
8. H/o Other Complaints (specify) …………………………………………………..
9. Family history of any liver disease…………………………………………………

**Clinical Examination (No -1 Yes -2)**

1. Diabetes (IDF criteria)………………………………………………………………..

If Yes, duration in months…………………………………………………….

1. HBS antigen + (by ELISA)………………………………………………………….
2. Anti HBc positivity …………………………………………………………………
3. Anti-HCV Positivity (by ELISA)……………………………………………………..
4. Co-infection of HIV (by ELISA)……………………………………………………..

## SPECIFIC DETAILS ABOUT LIVER DISEASE

1. CHILD/MELD score details
   1. Encephalopathy stage (0 if No encephalopathy)……………………………..
   2. Ascites (1-Absent 2-Mild, responsive 3-Moderate to Severe…………….

**•**

- 1. Serum Bilirubin (mg/dl) …………………………………………

**•**

- 1. Prothrombin time (INR) …………………………………………

**•**

- 1. Serum creatinine (mg/dL)…………………………………………
  2. Serum Albumin (mg/dL)…………………………………………

1. Chronic liver disease (Absent-0 Chronic hepatitis-1 Cirrhosis-2 HCC-3 ………

Carrier-4 Other-specify-5)

1. Etiology of CLD …………………………………………………………………..

HBV-1 HCV-2 HBV + HCV-3 Cryptogenic-4

Alcoholic-5 Autoimmune-6 Wilson’s dis.-7 Others- specify-8)

1. Date of first diagnosis of CLD (dd/mm/yy) ……………………
2. Acute liver disease (Absent-0 HAV-1 HEV-2 HBV-3 Drugs-4 ……………

Others-specify-5 Unknown-6)

**HOSPITALIZATION DETAILS**

1. Admitted as in-patient (No-1 Yes-2) ……………………………………….
2. If admitted, duration of hospital stay (days) …………………………………..
3. Discharge status (Alive-1 Died-2 LAMA-3 Transferred-4 ……….

Not admitted-5)

1. If died

**Cause: (No – 1 Yes-2)**

- 1. Variceal bleeding……………………………………………..
  2. Hepato-renal syndrome……………………………………….
  3. Hepatic encephalopathy………………………………………
  4. Sepsis………………………………………………………….
  5. HCC causing liver failure…………………………………….
  6. Other (specify) ……………………………………………….

**Date of death (dd/mm/yy)** ………………………

78. If not admitted, status (Recovered-1 No change-2 Complication-specify-3) …..

**Signature of the PI:**

**Date:**
