# Supplementary material for: Etiology and mode of presentation of chronic liver diseases in India: A multi centric study
Source: PLoS One. 2017 Oct 26;12(10):e0187033. doi: 10.1371/journal.pone.0187033 (PMC5658106; doi:10.1371/journal.pone.0187033)
Supplement: S3 Appendix — (PDF) [file pone.0187033.s003.pdf]

S3 Appendix:
Approval letter from Institutional Ethics Committee

Institutional Ethics Committee
Institute of Post Graduate Medical Education & Research
244, A.J.C. Bose Road, Kolkata-20.

Memo No. Inst./IEC/ 841

Date: 05.04.10

To
Dr. Kausik Das
Asst. Prof. Dept. of School of Digestive &
Liver Disease,
IPGME&R, Kolkata

A meeting of the Institutional Ethics Committee of IPGME&R, Kolkata was held on 27th March, 2010 at 12:00 noon. The following members were present:

Sl. No.	Name	Sex	Status	Affiliation/ Designation
1.	Prof. Subir Kumar Dutta	M	Chairman	Ex-Dean of Faculty of Medicine University of Calcutta & retired Professor of Pathology
2.	Prof. J. Chatterjee	M	Member	Head, NEMS
3.	Prof. Amal Santra	M	Member	Scientist, Gastroenterology
4.	Prof. Swati Chakraborty	F	Member	Head, Paed.-Medicine
5.	Prof. R.N. Dutta	M	Member	Head, Skin
6.	Prof. D. Bhattacharyya	M	Member- Secretary	Head, Pharmacology

The following project proposal submitted with other relevant documents were discussed:

"HCV: - THE INDIAN FACE".

List of other documents:

1. Forwarding Letter.
2. Patient Information Forms- English, Bengali, Hindi.
3. Patient Consent Forms- English, Bengali, Hindi.
4. Case Record Form

(Contd.Page-2)

Institutional Ethics Committee
Institute of Post Graduate Medical Education & Research
244, A.J.C. Bose Road, Kolkata-20.

After detailed deliberation and review, the following decision was taken:

☑ Approved
☐ Disapproved
☐ Resubmit after modification as detailed below
☐ Approved earlier but approval withdrawn now for reasons detailed below

☐ Reasons for disapproval ☐ Modifications suggested ☐ Reasons for withdrawing approval

Member-Secretary
Institutional Ethics Committee, IPGME&R, Kolkata-20.

Member - Secretary
INSTITUTIONAL ETHICS COMMITTEE
Institute of Post Graduate Medical Education
& Research. Kol-20
