# Supplementary material for: Etiology and mode of presentation of chronic liver diseases in India: A multi centric study
Source: PLoS One. 2017 Oct 26;12(10):e0187033. doi: 10.1371/journal.pone.0187033 (PMC5658106; doi:10.1371/journal.pone.0187033)
Supplement: S1 Table — (DOCX) [file pone.0187033.s004.docx]

S1 Table: Pair-wise comparison of Regions (P values) for Table 1

| Comparison | Illiteracy | Urban dwellers | Etiology | Age | Duration | BPL |
| --- | --- | --- | --- | --- | --- | --- |
| North vs East  North vs South  North vs West  North vs N-east  North vs Central | > 0.9  0.13  < 0.001  < 0.001  < 0.001 | < 0.001  < 0.001  < 0.001  < 0.001  < 0.001 | < 0.001  < 0.001  < 0.001  < 0.001  < 0.001 | 0.89  < 0.001  < 0.001  < 0.001  < 0.001 | < 0.001  < 0.001  < 0.001  < 0.001  < 0.001 | < 0.001  < 0.001  < 0.001  < 0.001  < 0.001 |
| East vs South  East vs West  East vs N-east  East vs Central | 0.01  < 0.001  < 0.001  < 0.001 | 0.04  < 0.001  < 0.001  < 0.001 | < 0.001  < 0.001  < 0.001  0.09 | 0.04  < 0.001  0.001  < 0.001 | < 0.001  0.09  < 0.001  < 0.001 | < 0.001  < 0.001  > 0.90  < 0.001 |
| South vs West  South vs N-east  South vs Central | < 0.001  0.45  0.30 | < 0.001  < 0.001  < 0.001 | 0.15  < 0.001  < 0.001 | < 0.001  0.60  < 0.001 | < 0.001  < 0.001  0.26 | < 0.001  > 0.90  < 0.001 |
| West vs N-east  West vs Central | < 0.001  < 0.001 | < 0.001  > 0.90 | < 0.001  < 0.001 | 0.11  0.99 | < 0.001  < 0.001 | < 0.001  < 0.001 |
| N-East vs Central | > 0.90 | < 0.001 | < 0.001 | 0.18 | < 0.001 | < 0.001 |
