# Supplementary material for: Etiology and mode of presentation of chronic liver diseases in India: A multi centric study
Source: PLoS One. 2017 Oct 26;12(10):e0187033. doi: 10.1371/journal.pone.0187033 (PMC5658106; doi:10.1371/journal.pone.0187033)
Supplement: S2 Table — (DOCX) [file pone.0187033.s005.docx]

S2 Table: Comparison of patients belonging to above and below poverty line

| Characteristic | Above Poverty Line  (N = 10299) | Below Poverty Line  (N = 2715) | P |
| --- | --- | --- | --- |
| Male | 7440 (72.4) | 2064 (76.0) | < 0.001 |
| Urban dweller | 5176 (50.3) | 1379 (50.8) | 0.62 |
| Illiterate | 683 (6.6) | 464 (17.1) | < 0.001 |
| Age at diagnosis | 42.9 ± 14.44 | 42.7 ± 14.43 | 0.59 |
| Drugs purchased OTC | 1253 (15.4) | 1381 (31.3) | < 0.001 |
| Etiology  HBV related  HCV related  HBV + HCV  Alcohol related  NAFLD related  Others | 3176 (30.8)  2454 (23.8)  56 (0.5)  1593 (15.5)  1544 (15.0)  1476 (14.3) | 1094 (40.3)  286 (10.5)  10 (0.4)  660 (24.3)  120 (4.4)  545 (20.1) | < 0.001 |
| Cirrhosis status  Non-cirrhotic  Compensated cirrhosis  Decompensated cirrhosis  Cirrhosis status not known  HCC | 6910 (67.1)  22 (0.2)  2876 (27.9)  134 (1.3)  357 (3.5) | 1253 (46.2)  4 (0.1)  1364 (50.2)  13 (0.5)  81 (3.0) | < 0.001 |
| Diabetes | 1277 (12.4) | 247 (9.1) | < 0.001 |
| Distance to nearest health  facility | 14.0 ± 82.34  2 (0 5) | 27.2 ± 153.00  3 (2 7) | < 0.001 |
| Availing Government  healthcare facility | 2722 (26.4) | 1393 (51.3) | < 0.001 |
