# Supplementary material for: Etiology and mode of presentation of chronic liver diseases in India: A multi centric study
Source: PLoS One. 2017 Oct 26;12(10):e0187033. doi: 10.1371/journal.pone.0187033 (PMC5658106; doi:10.1371/journal.pone.0187033)
Supplement: S3 Table — (DOCX) [file pone.0187033.s006.docx]

S3 Table: Break up of other etiologies mentioned in Table 1

| Serial No. | Etiology | Region | | | | | | Total |
| --- | --- | --- | --- | --- | --- | --- | --- | --- |
|  |  | North | East | South | West | Central | North-East |  |
| 1 | Unexplained Cholestatic Liver disease | 7 | 4 | 1 | 8 | 0 | 0 | 20 |
| 2 | Secondary biliary cirrhosis | 2 | 2 | 2 | 2 | 0 | 0 | 8 |
| 3 | Hepatic vein out flow obstruction | 72 | 49 | 18 | 20 | 7 | 1 | 167 |
| 4 | Wilson's disease | 16 | 35 | 19 | 6 | 4 | 1 | 81 |
| 5 | Unexplained liver functional abnormality | 266 | 662 | 207 | 176 | 84 | 44 | 1439 |
| 6 | Iron overload state due to chronic haemolytic disorders | 0 | 0 | 0 | 3 | 1 | 0 | 4 |
| 7 | Drug induced liver injury | 5 | 4 | 7 | 1 | 1 | 1 | 19 |
| 8 | Acute on chronic liver failure | 2 | 1 | 0 | 0 | 0 | 0 | 3 |
| 9 | Congestive hepatopathy | 0 | 0 | 2 | 20 | 1 | 0 | 23 |
| 10 | Liver hemangioma | 9 | 2 | 0 | 16 | 13 | 0 | 40 |
| 11 | Cystic diseases of liver | 25 | 4 | 1 | 15 | 1 | 1 | 47 |
| 12 | Hepatic adenoma | 2 | 0 | 0 | 1 | 0 | 0 | 3 |
| 13 | Metastatic tumors | 0 | 0 | 0 | 4 | 0 | 0 | 4 |
| 14 | Intrahepatic Cholangiocarcinoma | 1 | 0 | 0 | 7 | 1 | 0 | 9 |
| 15 | Extrahepatic Portal venous obstruction (EHPVO) | 26 | 6 | 22 | 30 | 1 | 0 | 85 |
| 16 | Hemochromatosis | 2 | 0 | 5 | 1 | 0 | 0 | 8 |
| 17 | Lymphoma | 1 | 0 | 0 | 3 | 3 | 0 | 7 |
| 18 | Unexplained hepato-splenomegaly | 20 | 16 | 2 | 13 | 2 | 1 | 54 |
|  | Total | 456 | 785 | 286 | 326 | 119 | 49 | 2021 |
